# Correlation between B7-H3 expression and matrix metalloproteinases 2 expression in pancreatic cancer

**DOI:** 10.1186/1475-2867-13-81

**Published:** 2013-08-16

**Authors:** Lihua Xu, Xiangmin Ding, Huo Tan, Jianjun Qian

**Affiliations:** 1 Center of Oncology and Hematology, The First Affiliated Hospital of Guangzhou Medical University, Guangzhou, Guangdong, 510230, China; 2 Department of Hepatopancreatobiliary Surgery, Subei People’s Hospital, Yangzhou, Jiangsu 225000, China

**Keywords:** B7-H3 expression, MMP-2, Pancreatic cancer, Prognosis

## Abstract

**Background:**

B7-H3 and matrix metalloproteinases 2 (MMP-2) are reported highly expressed in malignant tumor, we investigate the relationship between B7-H3 expression and MMP-2 on malignant behavior and prognosis predictable value in pancreatic cancer.

**Methods:**

We tested the expressions of B7-H3 and MMP-2 protein in 45 pancreatic surgical resected cancer samples; meanwhile, the clinicopathological data of enrolled patients were obtained for correlation analysis to obtain their relationship with pancreatic cancer progress.

**Results:**

The expression of B7-H3 was up-regulated with infiltrating depth, lymph node metastasis and TNM stage (P < 0.01). Positive expression rate of MMP-2 in pancreatic cancer tissues was 44.35%, whereas negative in normal pancreatic tissues. Multivariate analysis of Logistic regression showed B7-H3 and MMP-2 expressions were hazardous makers correlated with infiltrating depth (P < 0.05).

**Conclusion:**

Our study showed combined detections of B7-H3 and MMP2 protein expression could identify patients at high risk in disease recurrence and prognosis more efficiently.

## Introduction

Pancreatic cancer is one of the most aggressive and intractable cancers worldwide and a leading cause of cancer-related deaths [1]. The crude mortality rate of pancreatic cancer in China was 4.34% of the total cancer deaths [2]. It is calculated that, overall, pancreatic cancer is the fourth most common cause of cancer death, the overall survival of pancreatic cancer is <5% at 5 years. More than half of the patients have advanced disease with distant metastases at diagnosis [3]. The majority of pancreatic cancer is discovered at late stage and is therefore incurable, over the last decade several groups have embarked on screening individuals with recognized genetic syndromes or a significant family history for pancreatic cancer [4]. Although there is substantial diversity among the screened populations, these studies provide important information about the yield of these strategies [5].

B7-H3 (CD276) is a member of the B7 family and is expressed by lymphoid cells, such as dendritic cells, monocytes/macrophages and activated T cells; non-lymphoid tissue cells express B7-H3 too, such as epithelial cells, anterior pituitary progenitor cells, muscle cells and fibroblast-like synoviocytes [6,7], B7-H3 was reported expressed in non-small cell lung cancer, prostate cancer, neuroblastoma and renal cell carcinoma [8-10]. The expression of B7-H3 seems to correlate with clinicopathological features or poor prognosis [7,10], but there is one report demonstrating better survival in patients with gastric carcinoma B7-H3+ tumours [11]. These contradictory evidences suggest that single tumor marker is not sensitive or specific enough for tumor detection. Joint use of information from multiple markers may be more effective to reveal association between prognosis and data than single marker analysis.

The knock-down of B7-H3 lead to matrix metalloproteinase (MMP)-2, another tumor prognostic marker cell reduced in cell level [12]. MMPs (matrix metallo proteinases) are thought to play a major role on degrade all kinds of extracellular matrix proteins, and are regarded as a marker of malignant tumor invasion and metastasis [13-15]. Overexpression of various MMPs, particularly MMP-2, is correlated with poor prognosis in many types cancer including adrenocortical cancer, breast cancer, and thyroid malignancies [16,17].

There have been no studies conducted to elucidate the associations between MMP-2 and B7-H3 expression in pancreatic cancer prognosis. In present study, we analyzed the clinical significance of MMP-2 and B7-H3 expression in primary tumor samples resected from pancreatic cancer patients.

## Methods

### Patients

Pancreatic cancer tissue samples were obtained from patients who underwent pancreatic cancer surgery at our hospital from March in 2007. The study was approved by the ethics committee of Subei People’s Hospital, and all patients gave informed consent before surgery. Benign pancreatic tissues (confirmed by pathology) from operation were chosen as control.

TNM classification of these patients were recorded according to the proposed by the International Union against Cancer in 2003 [18]. Tissue samples were trimmed to proper volume and fixed in 10% formalin solution or stored at −80°C immediately. Histological examination was performed to verify the collected tissues in all samples.

### Quantitative RT-PCR for B7-H3

A total of 50 mg of −70°C frozen tissues were grind in liquid nitrogen bath. Extracts homogenized in 1 mL of buffer. After 15 min incubation on ice, the lysates were centrifuged at 12,000 rpm for 5 min at 4°C, and then the supernatants were removed. Precipitate resuspended in 100 μL B buffer, and shaken for 30 min at 4°C followed by centrifuge at 12,000 rpm for 15 min. 2 μL supernatants were mixed with 20 μL PCR solution buffer, and then reacted 30 min for extension at 30°C, 95°C for 10 min; followed 45 cycles of 95°C for 10 s, 63°C (55°C for GAPDH) for 20 s, and 72°C for 20s; a final extension at 55°C for 25 min, and a 4°C incubation.

Primer and probe sequences of B7-H3 and GAPDH were designed to assess the mRNA expression of each marker for RT-PCR. The forward primers, probe sequence and reverse primers for B7-H3 and GAPDH were: B7-H3 (forward), 5′ -GACAGCAAAGAAGATGATGGA- 3′; (probe), 5′-FAM-CCTCCCTACAGCTCCTACCCTCTGG- TAMRA-1-3′; (reverse), 5′-ACCTGTCAGAGCAGGATGC- 3′; GAPDH (forward), 5′-GGGTGTGAACCATGAGAAGT- 3′; (probe), 5′-FAM-CAGCAATGCCTCCTGCACCACCAA-TAMRA-1-3′; (reverse), 5′-GACTGTGGTCATGAGTCCT-3′, respectively. The integrity of the RNA was confirmed in a denaturing agarose gel using GAPDH as control.

All total RNA samples were reverse transcribed using the Advantage RT-for-PCR kit (Clontech Laboratories, Inc., Palo Alto, CA, USA). Samples were analyzed by quantitative RT-PCR (qRT-PCR) using the LightCycler System (Roche Diagnostics, Mannheim, Germany). B7-H3 mRNA copy numbers were normalized by GAPDH mRNA copy numbers (relative B7-H3 mRNA copies: absolute B7-H3 mRNA copies ⁄ absolute GAPDH mRNA copies).

### Immuno-histochemistry analyzing expression of B7-H3 and MMP-2 in tissues

The expression of B7-H3 proteins were evaluated in 45 tissue blocks of pancreatic cancer patients after histologically confirmed by immunohistochemistry. The semi-quantitative evaluation was conducted by Erkan M and co-workers’ method [19]. Quantification was made by the intensity of staining concluded by scores as follows: ① B7-H3positive cells / total cells in a microscope field scored as: ≤33% of the cancer cells: 1, >33 to ≤66% of the cancer cells: 2, >66% of the cancer cells: 3; ② intensity of staining-absent/weak: 1, moderate: 2, strong: 3. Each section had a final grade that derived from the multiplication of the area and intensity scores. Sections with a final score of ≤3 were classified as tumors with low B7-H3 expression (−), whereas sections with a final score of > 3 were classified as tumors with high B7- H3 expression (+).

MMP-2 expression was detected by Envision 2 step method [20]. Mouse anti human MMP-2 mono clone antibody and DAB developer were used in our study (ZeHao Bio Co. LTD.) Briefly, removed the slides out at −80°C, air dry at 60°C for about 30 min, and then dewaxed by regular steps. Washed by 1 × PBS, 30 sec, wet autoclave treatment for antigen 146 retrieval in Tris-EDTA, 3 min. Apply 3% H_2_O_2_, incubated 10 min at RT. Primary antibody was dilute to recommended concentration. The slides were incubated over night at 4°C, washed 3 × 5 min in PBS1x. Secondary antibody: Apply 1 drop Agent I, incubate 30 min at room temperature, washed 3 × 5 min in PBS, and then applied DAB solution, incubated for 10–15 min.

An image analyzer was used for quantification of immunoreactive of MMP-2, 10 selected fields of negative controls were chosen to compare. The mean values of the percentage of positive area for MMP-2 were recorded as each tumor expression.

### Survival analysis and statistical analysis

Kaplan-Meier survival curves were drawn to visually compare the survival rates of 45 pancreatic cancer patients in B7-H3 (+) MMP2 (+) group, B7-H3 (+) MMP2 (−) group, B7-H3 (−) MMP2 (+) group and B7-H3 (−) MMP2 (−) group at each TNM stage. The χ2 test, Fisher's exact probability and t tests were performed in SPSS 17.0 software for data analysis. Differences were considered statistically significant if P < 0.05.

## Results

### Demographic data of patients

A total of 45 pancreatic cancer tissue samples obtained successfully, 27 of them were males and 18 of them were females, their age ranged in from 48 to 64 years (mean, 56.3 years), Table 1 showed the detailed clinical data of these patients. The median follow-up period of these pancreatic cancer patients were 29.6 months (8-64 month). Another 27 cases of benign pancreatic tissues were chosen as control.

**Table 1:** Clinical data of 45 pancreatic cancer patients

**Cases number**	**Stage**	**Pathology type**			**Therapy**			
		**DA**	**AC**	**Others**	**RS**	**PS**	**RS + C**	**PS + C**
9	I	8	1		7	1	1	/
11	II	10	/	1	6	/	5	/
12	III	10	1	1	3	/	7	2
13	IV	9	2	2	/	1	/	12

*Abbreviation* used: *DA* Ductal Adenocarcinoma, *AC* Adenosquamous Carcinoma, *RS* Radical Surgery, *PS* Palliative Surgery, *C* Chemotherapy.

Chemotherapy including: Gemzar monotherapy, Gemzar + Oxaliplatin and Gemzar + Capecitabine.

### Highly expressed B7-H3 mRNA in pancreatic tumor tissues

The B7-H3 mRNA expression is significantly higher in 45 pancreatic tumor tissues than 27 benign pancreatic tissues (Table 2). In addition, the low 4- year survival rate of pancreatic cancer patients is correlated with high B7-H3 expression level in pancreatic tumor tissue.

**Table 2:** B7-H3 mRNA expression results of 45 pancreatic tumor tissue and 27 benign pancreatic tissues

	**B7-H3 mRNA expression**	**P value**
Normal	2.07 ± 0.60	0.000
Tumour	4.32 ± 2.05	
Without lymph nodes metastasis	2.59 ± 1.12	0.000
Lymph nodes metastasis	5.70 ± 1.48	
Survival <4 yr	4.89 ± 1.83	0.000
Survival >4 yr	2.04 ± 1.01	

### Low differentiated and metastatic patients have high B7-H3 protein expression

The positive expression rate of B7-H3 in pancreatic cancer tissue is 77.8% (35 of 45) and is negative in all benign tumor tissue. We summarized relationships between B7-H3 expression and clinicopathological results in Table 3. Positive B7-H3 expression is associated with low histo-differentiation, lymph node migration and TNM stages. The poor prognosis is correlated with higher positive B7-H3 in pancreatic cancer tissue but has no significant relationships with age, gender, tumor location, tumor size.

**Table 3:** The relationship between B7-H3 in pancreatic cancer tissue and associated clinical pathological factors

**Pathologic parameter**	**Cases(N)**	**B7-H3 expression positive rate %**	**χ**^**2**^	**P value**
Male	27	81.5 (22/27)	0.536	0.464
Female	18	72.2 (13/18)		
Age < 60	19	73.7 (14/19)	0.319	0.572
Age ≥60	26	80.8 (21/26)		
Located in head	37	81.1 (30/37)	0.257	0.612
Located in tail	6	66.7 (4/6)		
Whole pancreas cancer	2	50 (1/2)		
Size of primary carcinoma(<3 cm)	30	86.7 (26/30)	0.064	0.800
Size of primary carcinoma(≥3 cm)	15	60 (9/15)		
Histodifferentiation(II)	26	96.2 (25/26)	12.161	0.002*
Histodifferentiation(III)	7	57.2 (4/7)		
Histodifferentiation(IV)	12	50 (6/12)		
Lymph nodes metastasis Negative	20	65 (13/20)	16.071	0.000*
Lymph nodes metastasis Positive	25	88 (22/25)		
pTNM stage I	9	22.2 (2/9)	23.377	0.000*
pTNM stage II	11	72.7 (8/11)		
pTNM stage III	12	100 (12/12)		
pTNM stage IV	13	100 (13/13)		
Normal tissue	27	0 (0/27)		

*P < 0.01.

**Figure 1:**
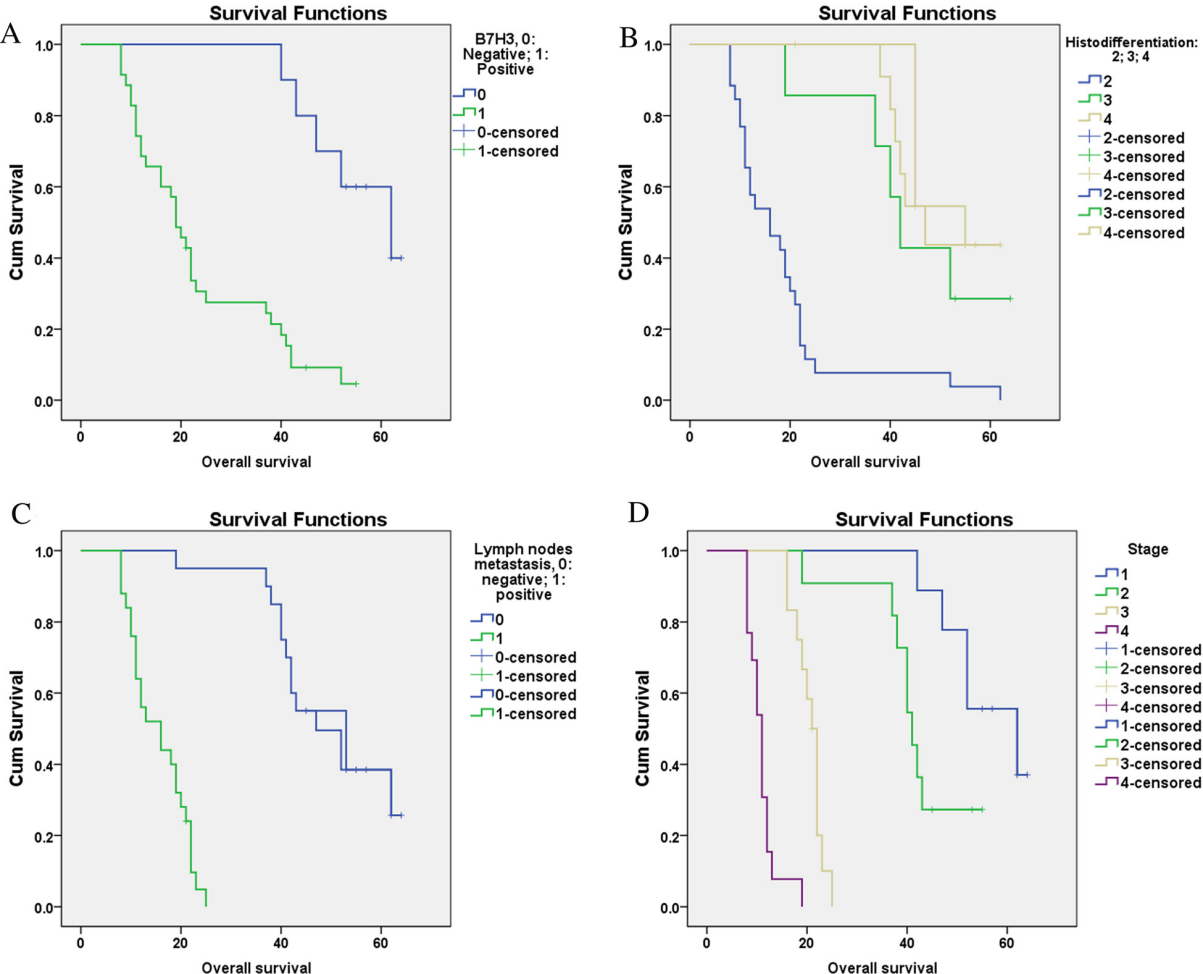
The comparative graphs of survival rate of B7-H3.

On the other hand, we observed negative B7-H3-expression patients have higher survival rate compared with positive patients (Figure 1), and the survival rate decreased when tumor stage increased, histo-differentiation decreased and lymph node migrated.

### Highly expressed MMP-2 in low differentiated and metastatic patients

**Figure 2:**
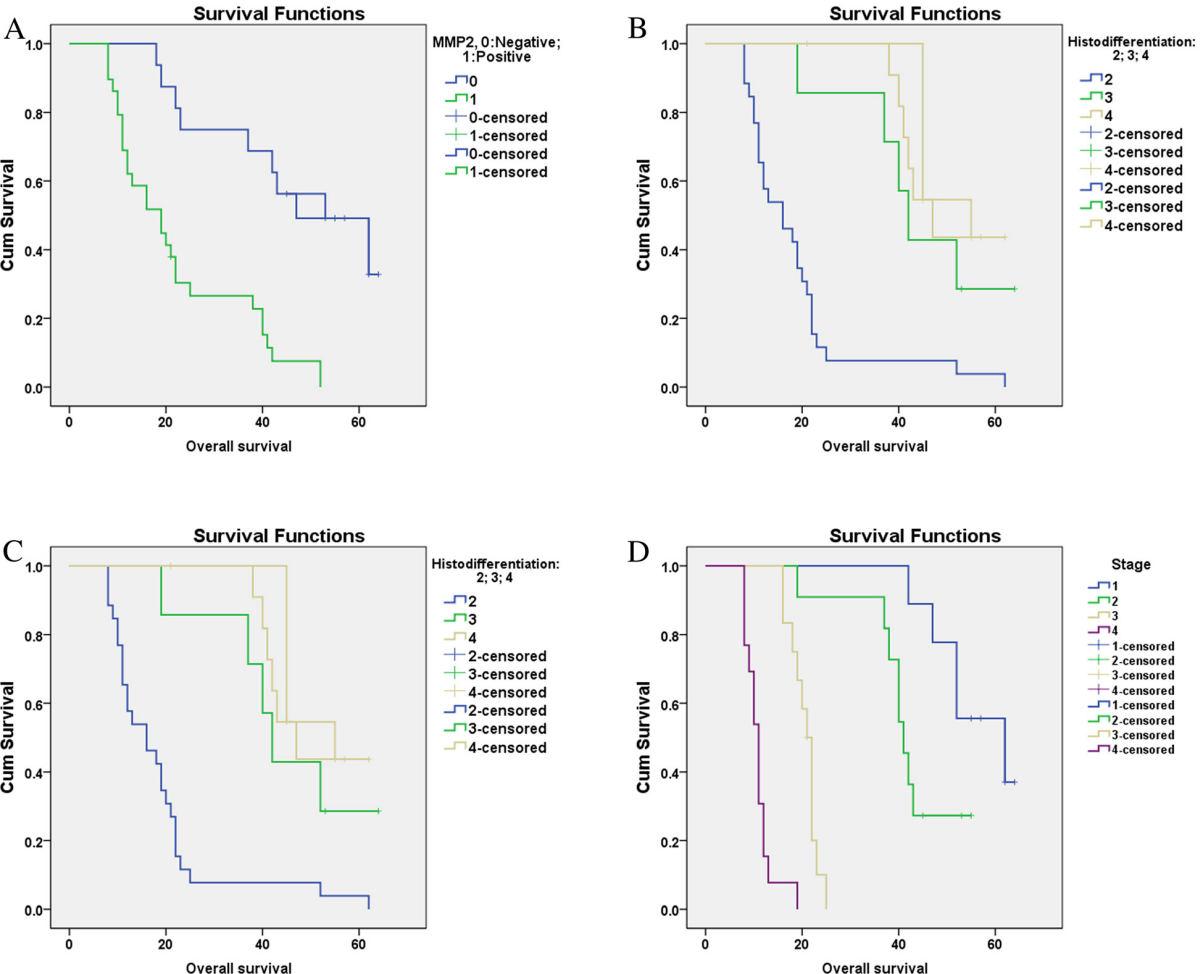
The comparative graphs of survival rate of MMP2.

As we can see from Table 4, when compared with low MMP-2 expression patients, the higher MMP-2 expression is, the worse prognosis of pancreatic cancer patients is (P = 0.001). Positive MMP-2 expression is associated with low histo-differentiation, lymph node migration and TNM stages but is not associated with other clinical characters. Figure 2 showed the survival function on KM estimator of MMP 2 in these pancreatic cancer patients.

**Table 4:** The relation between the expression of MMP-2 and clinic-pathological factors

**Pathologic parameter**	**Cases(N)**	**MMP2 expression positive rate %**	**χ**^**2**^	**P value**
Male	27	63.0 (17/27)	0.065	0.799
Female	18	66.7 (12/18)		
Age < 60	19	73.7 (14/19)	1.225	0.268
Age ≥60	26	57.7 (15/26)		
Located in head	37	67.6 (25/37)	0.886	0.642
Located in tail	6	50 (3/6)		
Whole pancreas cancer	2	50 (1/2)		
Size of primary carcinoma(<3 cm)	30	73.3 (22/30)	3.103	0.978
Size of primary carcinoma(≥3 cm)	15	46.7 (7/15)		
Histodifferentiation(II)	26	84.6 (22/26)	11.109	0.004*
Histodifferentiation(III)	7	43.9 (3/7)		
Histodifferentiation(IV)	12	33.3 (4/12)		
Lymph nodes metastasis Negative	20	35 (7/20)	13.621	0.000*
Lymph nodes metastasis Positive	25	88 (22/25)		
pTNM stage I	9	22.2 (2/9)	23.377	0.000*
pTNM stage II	11	45.5 (5/11)		
pTNM stage III	12	75 (9/12)		
pTNM stage IV	13	100 (13/13)		
Normal tissue	27	0 (0/27)		

*P < 0.01.

### B7-H3 expression is correlated with MMP-2 expression

Pearson correlation coefficients analysis for B7-H3 and MMP-2 showed a significant correlations in pancreatic patients (Pearson correlation = 0.496, P = .001), the increased B7-H3 expression is correlated with higher MMP-2 in pancreatic cancer samples (Table 5).

**Table 5:** Result of Pearson correlation coefficients analysis for MMP-2 and B7-H3

		**B7-H3**	**MMP2**
B7-H3	Pearson Correlation	1	.496*
	Sig. (2-tailed)		.001
	N	45	45

## Discussion

In this section, we described the prognosis significant of B7-H3 and MMP-2 expression alone and in combination in pancreatic cancer patients who underwent surgical resection. B7-H3 expression has been extensively studied in many kinds of malignant tumors for clinical diagnostic and/or prognostic utilities [6,10,21]. In patients with pancreatic cancer, the presence of B7-H3 expression has been shown to correlate with poor prognosis, Zhao et al. [22] detected that B7-H3 was significantly higher in pancreatic cancer tissue samples than that in normal pancreas tissues. Evidence also showed that B7-H3 may serve as an immunoinhibitory ligand which could promote tumor progression. For instance, B7-H3 ligand was shown on cell membranes of tumors obtained from children with advanced stage IV, but not early stage I neuroblastoma [23]. In a study of 70 patients with NSCLC, B7-H3 protein expressed was associated with an increased risk for tumor metastases [24]. In present study, elevated B7-H3 expression was detected in 77.8% of 45 pancreatic cancer cases.

In consistent with protein expression, we tested a significant increase of B7-H3 mRNA expression in pancreatic cancer tissue, like what was suggested by Arigami [10], that blood specimens from patients with gastric cancer contained significantly more copies of B7-H3 mRNA than those from healthy volunteers without cancer (P < 0.0001).

TNM classification is commonly accepted clinicopathologic predict prognosis for cancer patient. There have been several attempts to correlate B7-H3 expression with clinicopathologic variables, Takaaki et al. [10] found that B7-H3 expression was higher in gastric tumor cells than normal cells, and the expression was significantly correlated with overall stage. Some earlier studies also reported similar results [24]. Our present studies found that B7-H3 was more frequently elevated in pancreatic cancer tissue with lymph node involvement and tumor invasion. High B7-H3 expression was significantly associated with low 4-years survival rate. Higher B7-H3 expression was correlated with higher TNM stages, which indicates poor prognosis of the pancreatic cancer. But B7-H3 could not be used to accurately discriminate in age, gender, tumor location, size, stage. Therefore, B7-H3 expression analysis could be necessary,but not comprehensive for pancreatic cancer prognosis prediction. Combination test of B7-H3 expression with other carcinogenesis prognostic factors has been used for achieving more sensitive and precise prognostic prediction. Matrix metalloproteinases are a group of proteases that play important roles in the processes of tumor invasion and metastasis [13]. Recent studies have shown correlation of MMP2 and B7-H3 expression on tumor migration and immortality, Tekle et al. reported B7-H3 silencing of MDA-MB-435 cells resulted in reduced metastatic capacity, and significantly increased the median symptom-free survival of nude mice. Notably, the metastasis-associated proteins, matrix metalloproteinase (MMP)-2, signal transducer and activator of transcription 3 (Stat3), and the level of secreted interleukin-8 (IL-8) were reduced in the B7-H3 knock-down cell variants [12]. However, there has been no clear understanding of the prognosis significant of B7-H3 and MMP-2 in pancreatic cancer tissues. This is the first report to show a correlation between the levels of B7-H3 and the levels of MMP-2 in human pancreatic tumors, and these results suggest that MMP and B7-H3 are good predictors for pancreatic TNM stage.

The current research also has some shortcomings, and one of them is that the patient number in the subgroups is not high enough to draw a very solid conclusion, so further bigger population study is needed.

In conclusion, present study extends previous work; we demonstrated that combined analyzing B7-H3 and MMP-2 expression could predict prognosis of pancreatic cancer. Combining test increases the diagnostic significance over B7-H3 alone in pancreatic cancer patients. The predictive value using these biomarkers reported in this study is promising and warrants further validation in other prospective longitudinal cohort studies, preferably in general populations to avoid the potential patient selection bias inherent in most therapeutic/prevention trials.

## Competing interest

The authors declare that they have no competing interest.

## Authors’ contributions

LX was incharge of the design of whole experiment, carried out the statistical analysis job and drafted the manuscript. XD carried out real time PCR studies. HT carried out the IHC study. JQ are in charge of revise the manuscript and data analysis. All authors read and approved the final manuscript.
